# Evaluation of a new immunochromatographic assay for rapid identification of influenza A, B, and A(H1N1)2009 viruses

**DOI:** 10.1007/s10156-012-0533-1

**Published:** 2012-12-20

**Authors:** Keiko Mitamura, Chiharu Kawakami, Hideaki Shimizu, Takashi Abe, Yasushi Konomi, Yuki Yasumi, Masahiko Yamazaki, Masataka Ichikawa, Norio Sugaya

**Affiliations:** 1 Department of Pediatrics, Eiju General Hospital, 2-23-16 Higashi-Ueno, Taito-ku, Tokyo, 110-8645 Japan; 2 Yokohama City Institute of Health, Yokohama, Japan; 3 Kawasaki City Institute of Public Health, Kawasaki, Japan; 4 Abe Children’s Clinic, Yokohama, Japan; 5 Takahashi Clinic, Bando, Japan; 6 Yasumi Hospital, Morioka, Japan; 7 Zama Children’s Clinic, Zama, Japan; 8 Ichikawa Children’s Clinic, Isehara, Japan; 9 Department of Pediatrics, Keiyu Hospital, Yokohama, Japan

**Keywords:** Influenza virus, A(H1N1)2009, Rapid diagnosis, Immunochromatography

## Abstract

We evaluated Clearline Influenza A/B/(H1N1)2009, a new multi-line immunochromatographic assay for rapid detection of antigens of influenza A (Flu A), B (Flu B), and A(H1N1)2009 viruses. Clearline detected Flu A, Flu B, and A(H1N1)2009 viruses with a detection limit of 4.6 × 10^3^ to 7.5 × 10^4^ pfu/assay. The sensitivity and specificity of detection of influenza virus by Clearline, using RT-PCR as reference standard, were determined for A(H1N1)2009, Flu A, and Flu B, in nasopharyngeal aspirate, nasopharyngeal swab, and self-blown nasal discharge specimens. Sensitivity for nasopharyngeal aspirate specimens was: A(H1N1)2009 = 97.3 %, Flu A = 94.5 %, and Flu B = 96.8 %, and specificity was Flu A = 99.1 % and Flu B = 100 %. Sensitivity for nasopharyngeal swab specimens was: A(H1N1)2009 = 91.9 %, Flu A = 92.8 %, and Flu B = 100 %, and specificity was Flu A = 98.2 % and Flu B = 100 %. Sensitivity for self-blown nasal discharge specimens was: A(H1N1)2009 = 75.7 %, Flu A = 86.5 %, and Flu B = 76.2 %, and specificity was Flu A = 98.4 % and Flu B = 100 %. Sensitivity and specificity of Clearline were sufficient for nasopharyngeal aspirate and swab specimens. For self-blown nasal discharge specimens, sensitivity was lower than for nasopharyngeal aspirates and nasopharyngeal swabs. The sensitivity of Clearline for A(H1N1)2009 was good even 6 h after the onset of symptoms. These findings suggest that Clearline may be useful for early clinical diagnosis of influenza.

## Introduction

Seasonal and pandemic influenza are a major burden on health and the economy. New antivirals, for example neuraminidase inhibitors, have recently become available for management of influenza [1, 2]. Treatment and infection-control strategies are more effective when management is started at an early stage of illness and epidemics, so accurate and rapid diagnosis of influenza are very important for appropriate use of antivirals and for infection control. Although rapid influenza diagnostic tests (RIDTs) help early diagnosis of influenza and also detect A(H1N1)2009 virus as Flu A, RIDTs cannot distinguish A(H1N1)2009 from seasonal Flu A and the sensitivity of currently available RIDTs was reported to be lower than for viral culture or RT-PCR, especially during the A(H1N1)2009 virus pandemic [3–5]. In this study, we evaluated the clinical usefulness of the Clearline Influenza A/B/(H1N1)2009 test (Alere Medical, Tokyo, Japan), a new multi-line immunochromatographic assay for rapid detection of antigens of the Flu A, Flu B, and A(H1N1)2009 viruses.

## Materials and methods

### Basic evaluation

#### Detection limit

Ten vaccine strains and clinical isolates of influenza (Yokohama Institute of Public Health, Yokohama, Japan) [6] were used in this evaluation (Table 1). Viral titer was determined by plaque assays, and isolates were diluted serially in phosphate-buffered saline. A 100-μL aliquot of each viral suspension was mixed with 300 μL diluent, provided in the Clearline kit, and the mixture was then tested with Clearline. The detection limit was determined on the basis of Clearline results for different dilutions of each suspension.

**Table 1:** Detection limit of Clearline for influenza virus strains

Virus strain	Detection limit (pfu/assay)
A/California/7/2009 (H1N1)2009	5.3 × 10^4^
A/Yokohama/29/2010 (H1N1)2009	5.6 × 10^3^
A/Yokohama/1000/2009 (H1N1)2009	4.6 × 10^3^
A/New Caledonia/20/99 (H1N1)	5.8 × 10^4^
A/Yokohama/119/2009 (H1N1)	2.8 × 10^4^
A/Yokohama/22/2002 (H1N2)	3.2 × 10^4^
A/Hiroshima/52/2005 (H3N2)	2.1 × 10^4^
A/Yokohama/72/2010 (H3N2)	7.5 × 10^4^
B/Shanghai/361/2002 (Yamagata lineage)	1.7 × 10^4^
B/Malaysia/2506/2004 (Victoria lineage)	1.4 × 10^4^

### Clinical evaluation

#### Clinical specimens

Three-hundred and thirty nasopharyngeal swab specimens and 336 nasopharyngeal aspirate specimens were collected simultaneously from 336 patients, including both children and adults, with influenza-like illness (upper respiratory symptoms and/or fever) at two hospitals and four clinics (four pediatric, one internal medicine, and one pediatric/internal medicine) in Japan during the 2010/2011 influenza season (December 2010 to March 2011). Duration of fever was recorded by interview with patients. Another set of 268 patients with influenza like illness (upper respiratory symptoms and/or fever) was asked to blow nasal mucus into a collection film to furnish self-blown nasal discharge specimens. The results from the two patient cohorts were compared. The study protocol was approved by the ethics committee of Eiju General Hospital. All subjects provided informed consent to participate in this study.

Nasopharyngeal aspirate and self-blown nasal discharge specimens were tested with Clearline and with Espline influenza A&B–N (Fujirebio, Tokyo, Japan), without dilution by viral transport media. These specimens were sampled using swabs; the swabs were soaked in 1.5 mL viral transport medium and stored at −80 °C for RT-PCR as the reference assay. One nasopharyngeal swab collected from each patient was tested with Clearline.

#### Methods of measurement

##### Rapid diagnostic tests

Clearline Influenza A/B/(H1N1)2009 has three test lines, for differentiation of Flu A, Flu B, and A(H1N1)2009 viruses, and a control line [7]. Testing was performed in accordance with the manufacturer’s instructions. A specimen-collection swab provided in the kit was used to obtain the specimen. The swab containing the specimen was placed directly in a dedicated soft plastic tube containing 300 μL diluent provided in the kit. The swab was then rotated at least 5 times, squeezed against the interior wall of the tube, and pulled up while squeezing to extract the liquid from the swab. A test strip was inserted into the tube to start the reaction. After 10–15 min, the appearance of red–purple lines on the strip was assessed in accordance with the criteria in the manufacturer’s instructions.

Espline Influenza A&B–N is a lateral flow RIDT using enzyme immunoassay to differentiate Flu A and Flu B [8]. Testing was performed in accordance with the manufacturer’s instructions.

##### RT-PCR

The specimens were subjected to multiplex RT-PCR. A PCR sample was extracted from 200 μL of each stored suspension by use of the QIAamp^®^ Min Elute Virus Spin Kit (Qiagen, Valencia, CA, USA) in accordance with the manufacturer’s instructions. The multiplex RT-PCR was performed with Seeplex RV 12 ACE Detection kit and the combined Supplement procedure for detection and characterization of swine H1 influenza A virus provided by manufacturer (Seegene, Seoul, Korea), to detect A(H1N1) 2009, Flu A, and Flu B [9–11]. The HA gene of Flu A virus other than A(H1N1)2009 virus was detected, by use of real-time RT-PCR, to characterize subtype [12].

### Statistical analysis

SPSS statistics 20 (Japan IBM, Japan) was used for statistical analysis. A *p* value of less than 0.05 was considered statistically significant.

## Results

### Basic evaluation

#### Detection limit of Clearline

The detection limit of Clearline was 4.6 × 10^3^ to 7.5 × 10^4^ pfu/assay for ten influenza virus strains including A(H1N1)2009 (Table 1).

### Clinical evaluation

#### Sensitivity and specificity

Among the 336 nasopharyngeal aspirate specimens, multiplex RT-PCR detected A(H1N1)2009 virus in 149 specimens, Flu A strains other than A(H1N1)2009 in 73 specimens, and Flu B in 31 specimens. All Flu A strains other than A(H1N1)2009 were confirmed to be H3 strains (Flu A (H3)).

As Tables 2 and 3 show, the sensitivity of Clearline compared with multiplex RT-PCR for A(H1N1)2009, Flu A (H3), and Flu B was 97.3 % (145/149), 94.5 % (69/73), and 96.8 % (30/31), respectively. The specificity was 99.1 % (113/114) for type A influenza virus and 100 % (305/305) for Flu B. Among RT-PCR-confirmed A(H1N1)2009-positive specimens, nine samples were positive only on the A(H1N1)2009 test line of Clearline but negative on the Flu A test line. The corresponding sensitivity of Espline for A(H1N1)2009, Flu A (H3), and Flu B was 91.9 % (137/149), 94.5 % (69/73), and 100 % (31/31), respectively, and the specificity was 100 % (114/114) for type A influenza virus and 99.0 % (302/305) for Flu B.

**Table 2:** Comparison of Clearline and Espline with multiplex RT-PCR for 3 types of specimen

	Influenza detection by multiplex RT-PCR				
	Type A			Type B	
	(H1N1)2009 positive	A(H3) positive	Negative	Positive	Negative
Nasopharyngeal aspirate (*n* = 336)					
Clearline					
Positive	145	69	1	30	0
Negative	4	4	113	1	305
Total	149	73	114	31	305
Espline					
Positive	137	69	0	31	3
Negative	12	4	114	0	302
Total	149	73	114	31	305
Nasopharyngeal swab (*n* = 330)^a^					
Clearline					
Positive	137	64	2	31	0
Negative	12	5	110	0	229
Total	149	69	112	31	229
Self-blown nasal discharge (*n* = 268)					
Clearline					
Positive	78	32	2	16	0
Negative	25	5	126	5	247
Total	103	37	128	21	247
Espline					
Positive	75	36	0	16	0
Negative	28	1	128	5	247
Total	103	37	128	21	247

^a^Compared with multiplex RT-PCR results for nasopharyngeal aspirates

**Table 3:** Sensitivity and specificity of Clearline and Espline for 3 types of specimen

Specimen types	RIDTs	Sensitivity			Specificity	
		A(H1N1)2009	A(H3)	Type B	Type A	Type B
Nasopharyngeal Aspirate (*n* = 336)	Clearline	97.3 % (94.7–99.9)	94.5 % (86.6–98.5)	96.8 % (83.3–99.9)	99.1 % (97.4–100)	100.0 % (100–100)
	Espline	91.9 % (87.6–96.3)	94.5 % (86.6–98.5)	100.0 % (88.8–100)	100.0 % (100–100)	99.0 % (97.9–100)
Nasopharyngeal swab (*n* = 330)	Clearline	91.9 % (87.6–96.3)	92.8 % (83.9–96.3)	100.0 % (100–100)	98.2 % (95.8–100)	100.0 % (100–100)
Self-blown nasal discharge (*n* = 268)	Clearline	75.7 % (67.4–84.0)*	86.5 % (71.2–95.5)	76.2 % (52.8–91.8)	98.4 % (96.3–100)	100.0 % (100–100)
	Espline	72.8 % (64.2–81.4)*	97.3 % (85.8–99.9)	76.2 % (52.8–91.8)	100.0 % (100–100)	100.0 % (100–100)

Ranges in parentheses are 95 % confidence intervals

* p < 0.001: compared with both nasopharyngeal aspirate and swab specimens (chi-squared test)

Nasopharyngeal swabs were collected from 330 patients simultaneously with nasopharyngeal aspiration. Tables 2 and 3 show the performance of Clearline for nasopharyngeal swabs compared with results from multiplex RT-PCR of nasopharyngeal aspirates. The sensitivity of Clearline for A(H1N1)2009, Flu A (H3), and Flu B on nasopharyngeal swabs was 91.9 % (137/149), 92.8 % (64/69), and 100 % (31/31), respectively; specificity was 98.2 % (110/112) for type A influenza virus and 100 % (299/299) for Flu B.

Among 268 self-blown nasal discharge specimens, multiplex RT-PCR detected A(H1N1)2009 in 103 specimens, Flu A other than A(H1N1)2009 in 37 specimens, and Flu B in 21 specimens. All Flu A viruses other than A(H1N1)2009 were confirmed to be H3 strains (Flu A (H3)). Tables 2 and 3 list the performance of the assays for self-blown nasal discharge specimens. The sensitivity of Clearline compared with multiplex RT-PCR for A(H1N1)2009, Flu A (H3), and Flu B was 75.7 % (78/103), 86.5 % (32/37), and 76.2 % (16/21), respectively, and the specificity was 98.4 % (126/128) for type A influenza virus and 100 % (247/247) for Flu B. Among RT-PCR-confirmed A(H1N1)2009-positive specimens, eighteen specimens were positive on the A(H1N1)2009 test line of Clearline only but negative on the Flu A test line. The corresponding sensitivity and specificity for Espline compared with multiplex RT-PCR were almost same as for Clearline. For both Clearline and Espline, sensitivity for self-blown nasal discharge specimens for A(H1N1) 2009 was lower than for nasopharyngeal aspirate and nasopharyngeal swab specimens (*p* < 0.001 for each).

#### Effect of time after onset of fever on the sensitivity of Clearline

The effect of time after onset of fever on the sensitivity of Clearline and Espline is listed in Table 4 for nasopharyngeal aspirates and swabs confirmed positive by multiplex RT-PCR, for the 248 specimens for which time from onset of fever was known. The sensitivity of Clearline and Espline was more than 85 % for specimens collected more than 6 h after onset. Within 6 h of onset, the sensitivity of Clearline for A(H1N1)2009 was high (100 % for nasopharyngeal aspirate and 88.2 % for nasopharyngeal swab) although the sensitivity of Clearline for Flu A(H3) and of Espline for Flu A(H3) and A(H1N1)2009 was relatively low (80.0–82.4 %).

**Table 4:** Effect of duration of fever on sensitivity of Clearline and Espline (%)

Duration of fever (h)	Nasopharyngeal aspirate						Nasopharyngeal swab		
	Clearline			Espline			Clearline		
	A(H1N1)2009	Type A(H3)	Type B	A(H1N1)2009	Type A(H3)	Type B	A(H1N1)2009	Type A(H3)	Type B
~6	100 (17/17)	80.0 (8/10)	100 (3/3)	82.4 (14/17)	80.0 (8/10)	100 (3/3)	88.2 (15/17)	80.0 (8/10)	100 (3/3)
~12	95.7 (22/23)	85.7 (12/14)	100 (3/3)	91.3 (21/23)	92.9 (13/14)	100 (3/3)	91.3 (21/23)	92.9 (13/14)	100 (3/3)
~24	96.0 (72/75)	97.2 (35/36)	90.0 (9/10)	93.3 (70/75)	97.2 (35/36)	100 (10/10)	94.6 (71/75)	94.4 (34/36)	100 (10/10)
~48	100 (24/24)	100 (9/9)	100 (14/14)	95.8 (23/24)	100 (9/9)	100 (14/14)	91.7 (22/24)	100 (9/9)	100 (14/14)
49~	100 (7/7)	100 (2/2)	100 (1/1)	100 (7/7)	100 (2/2)	100 (1/1)	85.7 (6/7)	100 (2/2)	100 (1/1)

## Discussion

The performance of RIDTs for patients with influenza depends on viral load [13, 14]. Detection limits of currently available RIDTs have been reported to differ widely among viral strains or kits, ranging from approximately 10^3^ to 10^5^ pfu/assay or 10^3^ to >10^6^ TCID_50_/assay for seasonal influenza, A(H1N1)2009, and avian influenza [6, 15]. In this study, the detection limits of Clearline were approximately 10^4^ pfu/assay for Flu A and Flu B, and from 4.6 × 10^3^ to 5.3 × 10^4^ pfu/assay for A(H1N1)2009. The detection limit of Clearline was fairly consistent, irrespective of viral strain, and was equivalent to those previously reported for conventional diagnostic kits [6].

In our previously study, the sensitivity of Espline for nasopharyngeal aspirates compared with viral culture was 95.4 % (125/131) for Flu A (not including A(H1N1)2009) and 91.2 % (52/57) for Flu B [8]. Cheng et al. [13] reported low sensitivity of Espline for A(H1N1)2009(62 %), but the specimens in their study were diluted with viral transport medium. In our study, the sensitivity of Clearline and Espline for Flu A (H3) in nasopharyngeal aspirates using multiplex RT-PCR as reference standard was 94.5 % (69/73) and 94.5 % (69/73), respectively, which was high, and similar to that in our previous report. The specimens were tested directly, without dilution with viral transport medium. If test methods are adjusted, sensitivity of RIDTs may be sufficient for practical use. The corresponding figures for A(H1N1)2009 were 97.3 % (145/149) for Clearline and 91.9 % (137/149) for Espline. Although these values are not statistically significantly different, direct comparison shows Clearline was able to detect more A(H1N1)2009 cases than Espline (nine cases were positive by Clearline but negative by Espline, and one was negative by Clearline but positive by Espline) because of the high sensitivity of the A(H1N1)2009 test line of Clearline. Choi et al. [7] also reported relatively high sensitivity of the A(H1N1)2009 test line compared with the Flu A test line for nasopharyngeal swab specimens. If the A(H1N1)2009 line is negative for specimens with positive Flu A test line, this may suggest positive Flu A(H3) during interpandemics.

In this study, we collected two types of specimen simultaneously from 330 patients, and compared the sensitivity directly for nasopharyngeal aspirations and nasopharyngeal swabs. The sensitivity of Clearline was no different for nasopharyngeal swab and nasopharyngeal aspiration specimens.

Self-blown nasal discharge is a type of specimen recently approved for use in RIDTs in Japan. This type of specimen may reduce the burden of sampling—it is possible to collect specimens without medical equipment and from patients who must avoid injury to the nasal cavity. One limitation of this study is that self-blown nasal discharge specimens were obtained from a patient population different from that from which the nasopharyngeal aspirates and swabs were obtained, making direct comparison of sensitivity between specimen types somewhat more difficult. Although sensitivity of both Clearline and Espline for self-blown nasal discharges was more than 72 %, this was lower than for nasopharyngeal aspirates and swabs (*p* < 0.001). Physicians should be aware that the use of self-blown nasal discharge specimens may reduce the sensitivity of the assay, and should conduct specimen collection carefully to obtain an ample volume.

This study was conducted at four pediatric sites, one internal medicine site, and one pediatric/internal medicine site. Sensitivity and specificity were not statistically different among the 3 types of site. Further study may be necessary to determine whether patient age affects the sensitivity of RIDTs.

During the A(H1N1) influenza pandemic in 2009, deaths due to influenza were less common in Japan than in other countries. Use of anti-influenza agents from an early stage of infection is believed to be one reason for this lower mortality in Japan [16]. Analysis of Japanese pediatric patients hospitalized for respiratory disorders due to A(H1N1)2009 in this pandemic revealed 87.5 % developed dyspnea within 24 h of onset of fever [17]. It is recommended antivirals are started as soon as possible for patients with severe or progressive influenza, before the result from influenza testing is received [2]. It is, thus, important to promptly and correctly diagnose influenza and start treatment with anti-influenza agents [16]. However, there was a tendency toward lower accuracy of RIDTs on the first day of illness [5]. Our results showed sensitivity within 6 h of the onset of fever was relatively low for A(H1N1)2009 for Espline and for Flu A(H3) but that the sensitivity of Clearline for A(H1N1)2009 was high within 6 h of onset and for specimens collected after 6 h from onset. High sensitivity of the A(H1N1)2009 test line may contribute to the sensitivity within 6 h of onset of fever.

The cost of Clearline is equivalent to that of the other tests, when comparing the list prices (the price for Clearline is within the range of list price for other test, approximately US$ 11–16 per test; exchange rate ¥80/US$). So Clearline has the value of detecting the A(H1N1)2009 virus separately from the Flu A virus at a cost equivalent to those of the other conventional tests.

In conclusion, Clearline may be expected to help physicians to diagnose influenza promptly and detect A(H1N1)2009 virus separately from the Flu A(H3) virus in the clinical setting. Further studies may be necessary to evaluate the factors that affect the sensitivity of RIDTs.
